# The brain geography of GLP-1: An atlas for a new era

**DOI:** 10.61373/bm026d.0030

**Published:** 2026-03-24

**Authors:** Julio Licinio, Ma-Li Wong

**Affiliations:** 1 Genomic Psychiatry, Genomic Press, New York, New York 10036, USA

Why do large molecules that cannot cross the blood-brain barrier (BBB), such as hormones or immune mediators, have significant effects on the brain? There are many examples. During food intake, right after cholecys-tokinin (CCK) is produced in the gut, the brain soon afterward synthesizes its own CCK (1, 2). In 1997, while working in Bethesda at the National Insti-tutes of Health, we showed that a single injection of bacterial endotoxin into a rat’s abdomen caused the brain to activate IL-1*β* gene expression in the hypothalamus, the circumventricular organs, the choroid plexus, and throughout brain parenchyma (3). Our conclusion nearly thirty years ago was that the brain has its own inflammatory response and, in stark con-trast to what happens at peripheral sites, executes it with almost no reg-ulation by anti-inflammatory cytokines. The peripheral immune system ignited the process, and then the brain fueled the fire. We showed that these immune mediators did not need to be imported from the periphery; they were made within the brain. We also found the same pattern with other inflammatory mediators, such as nitric oxide (NO) and its inducible enzyme, NO synthase 2 (NOS2) (4, 5). Why is this principle relevant now?

As more and more people inject themselves with drugs based on GLP-1 (6), we overlook the fundamental fact that GLP-1 survives for only about one to two minutes in the bloodstream (7). Two minutes! Whatever the gut secretes after a meal is rubble by the time it reaches the cortex. So the brain, characteristically, has arranged its own supply chain. Pre-proglucagon neurons in the nucleus of the solitary tract. A smaller popu-lation in the olfactory bulb. These cells produce GLP-1 for central use, and they project widely.

Now, semaglutide is a 26-billion-dollar-per-year drug. With annual revenues of 6 billion dollars, liraglutide is not far behind (8, 9). The brain is now a major capital target (10). People are losing weight, yes, but they also report that they have stopped thinking about alcohol and craving cigarettes (11, 12). Clinical trials for depression (13) and Alzheimer’s dis-ease (14) are underway or being planned. The central effects of these compounds are clearly profound and not limited to appetite. Yet, until now, no one has produced a proper atlas of where the brain actually makes this peptide, and whether there are sex differences in its brain geography.

Ryu, Gumerova, Pevnev, Yuen, and Zaidi achieved exactly this, and we were pleased to see it (15). Their paper in this issue of Brain Medicine uses RNAscope, which detects single mRNA transcripts, to map Glp1 expres-sion across 25 brain regions in male and female mice (Figure 1). Three animals per sex. Every tenth section. Two blinded observers counted by hand. Painstaking, old-fashioned neuroanatomy combined with a molec-ular technique of exceptional sensitivity.

## What did they find?

First, let us quickly review what was expected: findings at the level of the solitary tract and the olfactory bulb. Yes, the highest concentrations were where the literature predicted them to be. However, Ryu et al. reveal un-expected sex differences: females had higher *Glp1* densities in the raphe obscurus and in the ventral and ventrolateral parts of the solitary nucleus, while males, interestingly, showed significantly more *Glp1* in the granu-lar cell layer of the olfactory bulb. Several hindbrain nuclei expressed *Glp1* exclusively in one sex or the other; for example, the ambiguus nucleus only in females and the central subnucleus of the solitary tract only in males. But the pattern is far from random and closely matches clinical observa-tions: GLP-1 analogs tend to suppress appetite more effectively in women, leading to greater weight loss. The glycemic benefits similarly favor fe-males. As in any study, there are limitations, which the authors clearly recognized, including small sample sizes and the fact that females were not staged for the estrous cycle. Additionally, RNAscope detects mRNA, not peptides or functions. These are real constraints that limit what this initial atlas can demonstrate at this stage. However, they do not diminish what it reveals.

## Why? Nobody knew, exactly. Now there is at least a map suggesting where to look

The authors go further. We were thrilled to follow them as they traced *Glp1*’s involvement with estrogen receptor signaling, serotonergic neu-rons in the raphe, and the NPY-POMC axis that regulates hunger and sati-ety in the arcuate nucleus. The emerging picture is not of a single peptide acting alone but of a coordinated group, sexually dimorphic at every level, with GLP-1 as one of several instruments, perhaps the most prominent, given the pharmacological power now directed at its receptor. But this was not a soliloquy. Upon first seeing this paper, we rapidly concluded: yes, as is the case with CCK (2), the brain does not need to borrow GLP-1 from the gut. It runs a parallel operation, autonomous, locally regulated, and organized along sexual lines that mirror the clinical pharmacology of drugs currently prescribed to millions of humans. We showed nearly thirty years ago that the brain does exactly this with inflammatory cytokines. The principle is conserved: peripheral signal, central recapitulation, inde-pendent regulation. What changes is the molecule; what remains constant is the principle of distinct peripheral and central compartments that are integrated but possibly regulated differently.

Ryu and colleagues have provided us with a blueprint for an innova-tive branch of that architecture. We have always found that the gap from a mouse atlas to a human therapy is far from trivial. But you cannot close a gap you have not yet measured, and you cannot measure it without know-ing where the journey begins. Let us open the first atlas and take the first steps on this remarkable journey of a thousand miles.

## Figures and Tables

**Figure 1.**
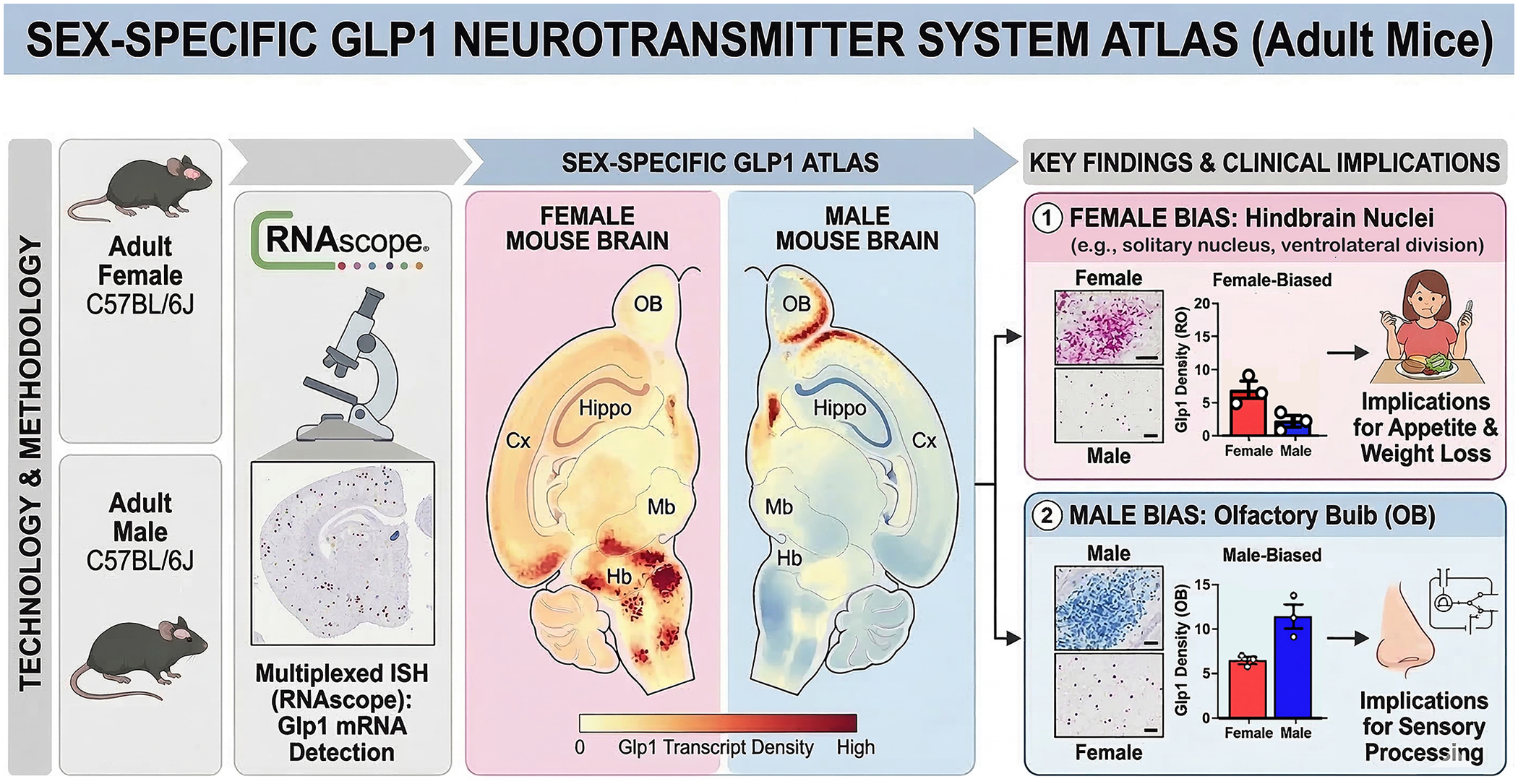
Sex-Specific Spatial Atlas of *Glp1* Expression in the Adult Mouse Brain. This schematic shows sex-biased *Glp1* mRNA distribution detected via RNAscope in situ hybridization in adult C57BL/6J mice. Female brains display higher *Glp1* transcript densities in hindbrain nuclei (e.g., solitary nucleus, ventrolateral), suggesting enhanced appetite suppression and weight loss effects. Males show greater densities in the olfactory bulb granular layer, indicating possible roles in sensory processing. Key clinical implications include potential sex differences in GLP-1 analog effectiveness for metabolic and psychiatric disorders. Credit: Initial conceptualization and structural layout of this figure were assisted by Gemini (Google), with final rendering and extensive manual reconstruction performed by the authors.
